# Selective association of plasma sphingolipid species with insulin sensitivity and secretion in normoglycemic Black and White American adults

**DOI:** 10.3389/ebm.2025.10538

**Published:** 2025-06-24

**Authors:** Peace Asuzu, Naser Aliye Feto, Jim Wan, Frankie Stentz, Nawajes Mandal, Samuel Dagogo-Jack

**Affiliations:** ^1^ Department of Medicine, Division of Endocrinology, Diabetes and Metabolism, University of Tennessee Health Science Center, Memphis, TN, United States; ^2^ Department of Ophthalmology, University of Tennessee Health Science Center, Memphis, TN, United States; ^3^ Department of Preventive Medicine, University of Tennessee Health Science Center, Memphis, TN, United States

**Keywords:** adiposity, beta-cell function, ceramide, insulin resistance, sphingomyelin

## Abstract

Ceramides and other sphingolipids are associated with diabetes risk. Here, we examined the association of plasma sphingolipids with insulin sensitivity and secretion in people without diabetes. We enrolled adults without diabetes based on 75-g oral glucose tolerance test. Assessments included clinical examination, insulin sensitivity (hyperinsulinemic euglycemic clamp), and insulin secretion (intravenous glucose tolerance test). Plasma levels of 58 sphingolipid species (including ceramides, monohexosylceramides, sphingomyelins, and sphingosine) were assayed using liquid chromatography tandem mass spectrometry. The study participants (N = 240; 129 Black, 111 White) had a mean age of 43.1 ± 12.0 y, body mass index (BMI) 29.4 ± 6.23 kg/m^2^, fasting plasma glucose 91.4 ± 6.91 mg/dL, and 2-h plasma glucose 123 ± 26.3 mg/dL. Several of the 58 SPLs species assayed showed variable associations with insulin sensitivity (r = 0.17–0.35, P = 0.039 - <0.0001) and secretion (r = 0.14–0.27; P = 0.038 - <0.0001). After correction for multiple testing, plasma levels of very-long-chain (VLC) monohexosylceramide C34:0 (r = 0.31 – 0.43, P < 0.0001) and VLC sphingomyelins C28-C34 (r = 0.31–0.35, P = 0.0004 - <0.0001) were significantly associated with insulin sensitivity. Plasma VLC sphingomyelin level were inversely associated with insulin secretion, plasma glucose, BMI, and waist circumference. We conclude that circulating VLC sphingomyelins are associated positively with insulin action and inversely with insulin secretion and adiposity in normoglycemic adults, indicating a possible link to glucoregulation that precedes the development of dysglycemia.

## Impact statement

Accumulation of ceramides and other sphingolipids results in lipotoxicity, insulin resistance, and increased diabetes risk. We assessed the relationship between circulating levels of selected sphingolipids and glucoregulatory physiology in adults without diabetes. Our findings demonstrate heterogeneity among plasma sphingolipid species regarding their association with sensitivity and secretion. Long-chain ceramides and sphingomyelins associate inversely with insulin sensitivity and positively with insulin secretion; very-long-chain sphingolipids species associate positively with insulin sensitivity and inversely with insulin secretion. Very-long-chain sphingolipids also were inversely associated with adiposity and glycemia, indicating a beneficial glucoregulatory and metabolic profile. These findings suggest a physiological link between metabolism of certain sphingolipids and glucose regulation long before the development of dysglycemia.

## Introduction

Sphingolipids are specialized lipids in mammalian cells that have a structure characterized by the presence of an amino dihydroxy alkane, a sphingoid base, an N-bound fatty acid, and a variable head group [1–3]. The major classes of sphingolipids include ceramides, monohexosylceramides, sphingomyelins, and sphingosines, among others. Variability in carbon length, saturation, and hydroxylation within structural components of sphingolipids has generated several thousand individual species of sphingolipids [1–3]. The spectrum of biological roles of sphingolipids includes cell membrane integrity and modulation of inflammation, signaling, differentiation, growth, senescence, and cell death [4–7].

Tissue accumulation of ceramides impairs insulin signaling and induces pancreatic beta-cell apoptosis, with consequent glucose dysregulation [7–9]. Furthermore, the roles of specific sphingolipids in the pathophysiology of diverse endocrine and metabolic conditions, including mitochondrial dysfunction, thyroid eye disease, autoimmune disorders, metabolic dysfunction-associated fatty liver disease, brain insulin resistance and neurodegenerative disorders, and glucose dysregulation are being increasingly recognized (reviewed recently by Li et al. [7]).

Modern lipidomic technology has enabled precise quantitation of individual sphingolipid species in human plasma, and alterations in circulating levels of certain sphingolipid species have been associated with obesity, diabetes, and prediabetes [10–15]. Furthermore, plasma levels of specific ceramides and other sphingolipid species have been reported to be significantly associated with measures of insulin sensitivity and insulin secretion in studies that included individuals with obesity or type 2 diabetes [9, 16–18]. Hyperglycemia is known to increase *de novo* synthesis of ceramides and alter the abundance of other sphingolipid species through modulation of specific enzymes involved in the sphingolipid metabolism, including serine palmitoyltransferase and sphingomyelinases [19–21].

The aim of the present report was to determine the association of a broad range of circulating sphingolipid species with measures of insulin sensitivity and insulin secretion in individuals without diabetes. By studying such individuals, we hoped to avoid any metabolic effects of dysglycemia *per se* on lipid metabolism [19–21]. The development of insulin resistance precedes the occurrence of type 2 diabetes by several years; similarly, the loss of first-phase insulin secretory response to glucose challenge represents early evidence of pancreatic beta-cell defect that precedes the diagnosis of diabetes by several years [22, 23]. Thus, we reasoned that the reports linking sphingolipids to diabetes probably represent a distal relationship that might be discernible proximally, prior to the occurrence of dysglycemia. The aim of the present study was to assess the association of plasma levels of 58 selected sphingolipid species with insulin sensitivity and insulin secretion in adults without diabetes.

## Materials and methods

### Study subjects

Study participants were selected from the Pathobiology of Prediabetes in a Biracial Cohort (POP-ABC) [24, 25]. Participants were eligible for the POP-ABC study if they were 18–65 years, self-reported as non-Hispanic Black (African American) or non-Hispanic White (European American) and had biological parent(s) with type 2 diabetes. Additional participants were included from the POP-ABC normative cohort comprising African American European American adults without a family history of diabetes. All participants underwent a screening 75-gram oral glucose tolerance test (OGTT) to document normal fasting plasma glucose (FPG; <100 mg/dL [5.6 mmol/L]) and/or normal glucose tolerance (2-h plasma glucose [2hPG] <140 mg/dL [7.8 mmol/L]) [17, 18]. Intercurrent illness, hyperglycemia, a history of diabetes, or exposure to medications known to alter glucose metabolism were among the exclusion criteria [24, 25]. A written informed consent was obtained from all participants before enrollment in the POP-ABC study [24, 25].

For the present report, we selected African American participants (N = 129) and European American participants (N = 111), matched in age (within 5 years) and sex, who had completed assessment of insulin sensitivity and insulin secretion at enrollment [20, 21]. Plasma specimens obtained from the participants at enrollment were analyzed for sphingolipid levels in the ongoing Ceramides and Sphingolipids as Predictors of Incident Dysglycemia (CASPID) study. The CASPID study protocol (IRB Approval #21-07936-FB) was approved by the University of Tennessee Health Science Center (UTHSC) Institutional Review Board and carried out at the UTHSC General Clinical Research Center (GCRC).

### Assessments

Participants arrived at the GCRC after fasting overnight for baseline assessments. These included a structured medical interview and general physical examination, measurement of height, weight, waist circumference, and OGTT. The body mass index (BMI) was calculated as weight in kilograms divided by the height in meters squared. Additional assessments included measurement of insulin sensitivity and insulin secretion.

### Oral glucose tolerance test

Ahead of the baseline visit, participants received written instructions to continue with their normal diet, refrain from strenuous exercise and alcohol consumption for a day prior, and to avoid smoking the morning of the OGTT. After an overnight fast, venous blood specimens were sampled from participants before (0 min) and at 30 and 120 min after drinking 75 g of flavored glucose solution (Trutol 75; Custom Laboratories, Baltimore, MD) [24, 25].

### Insulin sensitivity

Whole body insulin sensitivity was assessed using the hyperinsulinemic euglycemic clamp method of DeFronzo et al. [26]. In brief, participants were fasted overnight for approximately 12 h before undergoing the clamp studies at the GCRC. A primed, continuous intravenous infusion of regular insulin (2 mU/kg/min; 12 pmol/kg/min) was administered for 180 min through intravenous cannulas in both arms, while maintaining blood glucose level at ∼100 mg/dL (5.6 mmol/L) with a variable rate dextrose (20%) infusion. Glucose and insulin measurements were carried out on arterialized blood samples obtained every 10 min during the procedure. During the final 60 min of insulin infusion (steady state), the total insulin-stimulated glucose disposal rate (M) was calculated and corrected for the steady-state plasma insulin levels to derive the final insulin sensitivity index (Si-clamp), as previously described [24–26]. Fasting glucose and insulin levels were used to calculate the homeostasis model assessment of insulin resistance (HOMA-IR) (HOMA2 calculator. https://homa-calculator.informer.com/2.2/) based on Matthews et al. [27].

### Insulin secretion

Acute insulin secretory response to glucose (AIRg) was assessed using the frequently sampled intravenous glucose tolerance test (FSIGT), as previously described [24, 25]. In brief, after an overnight fast, participants arrived at the GCRC for the FSIGT. An intravenous dextrose bolus (25g) was administered, with arterialized blood sampling for the measurement of glucose and insulin levels at −30, 0, 2, 3, 4, 5, 7, and 10 min relative to dextrose bolus. The AIRg was computed as the mean incremental insulin concentration from 3 to 5 min after the dextrose bolus [23, 25, 28]. Fasting glucose and insulin levels were used to calculate the homeostasis model assessment of beta cells (HOMA-B) (HOMA2 calculator. https://homa-calculator.informer.com/2.2/) based on Matthews et al. [27]. The disposition index, a measure of the insulin secretion corrected for ambient insulin sensitivity, was calculated as AIRg x Si-clamp [29].

### Biochemical assays

Plasma glucose levels during OGTT and hyperinsulinemic euglycemic clamp were measured at the bedside using the YSI glucose analyzer (Yellow Spring Instruments Co., Inc., Yellow Spring, OH). Plasma insulin levels were measured in our Endocrine Research Laboratory with a solid phase, two-site sequential chemiluminescent immunometric assays on the Siemens Immulite analyzer (Siemens Healthcare Diagnostics Products, Ltd., Camberley, Surrey, UK). The within-batch variation coefficient for the insulin assay was <5%.

### Lipidomic analysis

Targeted lipidomic analysis was performed in baseline plasma specimens obtained at enrolment in the POP-ABC study. The plasma specimens preserved in EDTA were stored at −80°C until the lipidomic analysis. A total of 58 selected sphingolipids of various classes (ceramides, monohexosyl-ceramides, sphingomyelins, sphingosine and dihydro-sphingosine-1-phosphate) were assayed using liquid chromatography-tandem mass spectrometry (LC-MS/MS). The measurements were carried out at the Lipidomics Core at Virginia Commonwealth University, Richmond, Virginia, using established protocols [19, 20]. The individual sphingolipid species were validated and quantified using a Shimadzu LC-20 AD binary pump system coupled to a SIL-20AC autoinjector, and a DGU20A3 degasser coupled to an ABI 4000 quadrupole/linear ion trap (QTrap) (Applied Biosystems), operating in triple quadrupole mode. Using the retention times and m/z ratio, the 58 SPL species were identified and quantified by comparing the target lipid ion of interest with the normalization of quantitated ion abundances [30, 31].

### Statistical analysis

Data were reported as means ± SD. The significance of differences between defined groups was analyzed using t-test for continuous variables and chi squared test for categorical variables. Linear regression models were used to assess the relationship between individual sphingolipid species and measures of adiposity, insulin secretion, and insulin sensitivity. Significance level was generally set as P < 0.05 (two-tailed). For analysis of the association of 58 individual sphingolipid species with insulin sensitivity or insulin secretion, a Bonferroni-corrected significant P value of <0.0009 was adopted, to minimize false discovery from multiple comparisons. The analyses were performed using SAS 9.4 (SAS Institute Inc., Cary, NC).

## Results

### Cohort description

The study population (N = 240; 150 women, 90 men) comprised 129 African American (non-Hispanic Black) and 111 European American (non-Hispanic White) participants. Table 1 shows their baseline characteristics. The mean age was 43.1 ± 12.0 y, BMI 29.4 ± 6.23 kg/m^2^, fasting plasma glucose 91.4 ± 6.91 mg/dL, and 2-hr plasma glucose 123 ± 26.3 mg/dL (Table 1). The mean fasting plasma glucose was lower, and the BMI and waist circumference were higher, in African American vs. European American participants. However, there were no ethnic differences in 2-hr plasma glucose or total plasma levels of sphingolipids, ceramides, monohexosyl ceramides, sphingomyelins, and sphingosine (Table 1).

**TABLE 1:** Baseline characteristics of study participants.

Characteristics	All	African American	European American	P-value
Number	240	129	111	
Male/Female	90/150	48/81	42/69	
Age (yr)	43.1 ± 12.0	42.0 ± 11.6	44.4 ± 12.4	0.12
BMI (kg/m^2^)	29.4 ± 6.23	30.9 ± 6.52	27.7 ± 5.40	<0.0001
Female	30.2 ± 6.49	32.0 ± 6.31	28.1 ± 6.08	0.0002
Male	28.2 ± 5.59	29.2 ± 6.55	27.1 ± 4.02	0.075
Waist circum. (cm)	93.4 ± 15.0	96.2 ± 15.5	90.1 ± 13.8	0.0017
Female	92.0 ± 15.1	95.8 ± 14.8	87.5 ± 14.3	0.0007
Male	95.7 ± 14.6	96.9 ± 16.6	94.4 ± 11.7	0.42
FPG (mg/dL)	91.4 ± 6.91	90.3 ± 7.12	92.5 ± 6.50	0.013
2hrPG (mg/dL)	123 ± 26.3	123 ± 25.9	123 ± 26.9	0.93
Total sphingolipids (nmol/mL)	96.0 ± 25.7	96.3 ± 26.0	95.7 ± 25.4	0.85
Total ceramides (nmol/mL)	3.36 ± 1.48	3.22 ± 1.42	3.53 ± 1.54	0.11
Monohexosyl ceramides (nmol/mL)	4.62 ± 2.73	4.45 ± 2.78	4.82 ± 2.66	0.28
Total sphingomyelins (nmol/mL)	87.4 ± 23.7	88.0 ± 23.9	86.8 ± 23.7	0.68
Sphingosine (pmol/mL)	121 ± 54.8	126 ± 54.3	116 ± 55.1	0.18

FPG, fasting plasma glucose; 2hrPG, two-hour plasma glucose. To convert plasma glucose from mg/dL to mmol/L, multiply by 0.056.

### Association of selected plasma sphingolipids with insulin sensitivity

Nominally significant associations were observed between several sphingolipid species and whole-body insulin sensitivity (Si-clamp) (Supplementary Table S1). Plasma levels of long-chain ceramide C18:1 (r = −0.22, P = 0.0062) and long-chain sphingomyelin C18:1 (r = −0.23, P = 0.0046) were inversely associated with insulin sensitivity (Supplementary Table S1). In contrast, plasma levels very-long-chain ceramide C26:0 (r = 0.20, P = 0.014), monohexosylceramide C34:0 (r = 0.30, P = 0.0002) and sphingomyelin C28 – C34 species were positively associated with insulin sensitivity (Supplementary Table S1). No other circulating sphingolipid species (including ceramides, monohexosylceramides, sphingomyelins or sphingosines) reached even nominally significant association with insulin sensitivity in our study population.

After correction for multiple testing, plasma levels of very-long-chain monohexosylceramides C34:0 (r = 0.30, P 0.0002), very-long-chain sphingomyelins C28:1, C28:0, C30:1, C30:0, C32:0, and C34:0 (r = 0.31–0.35, P = 0.0004 - <0.0001) were positively associated with insulin sensitivity (Table 2; Supplementary Table S1). Notably, there was no significant association between plasma levels of total ceramides, total monohexosylceramides, sphingosine, or total sphingolipids and insulin sensitivity (Supplementary Table S1). In contrast, plasma levels of total very-long-chain sphingomyelins were significantly associated with insulin sensitivity (r = 0.35, P < 0.0001) (Supplementary Table S1).

**TABLE 2:** Plasma sphingolipid species significantly associated with insulin sensitivity or insulin secretion.

Insulin sensitivity				
Sphingolipids (pmol/mL)	Mean ± SD	r	P Value	Adjusted P value^a^
MHC C34:0	0.28 ± 0.28	0.30	0.0002	0.0047
SM C28:1	18.5 ± 8.07	0.34	<0.0001	0.0003
SM C28:0	39.3 ± 20.7	0.31	0.0001	0.0003
SM C30:1	10.1 ± 4.39	0.29	0.0004	0.0026
SM C30:0	8.35 ± 3.89	0.32	<0.0001	0.0003
SM C32:0	2.74 ± 1.49	0.35	<0.0001	0.0002
SM C34:0	1.22 ± 0.61	0.31	0.0001	0.0023
Total VLC	82.5 ± 36.2	0.35	<0.0001	<0.0001

Insulin secretion				
Sphingolipids (pmol/mL)	Mean ± SD	r	P Value	Adjusted P value
SM C28:0	39.3 ± 20.7	−0.25	0.0003	0.0012
SM C30:1	8.35 ± 3.89	−0.27	<0.0001	0.0007

^a^ Adjusted for age, sex, race/ethnicity and BMI.

### Association of selected plasma sphingolipids with insulin secretion

Compared to insulin sensitivity, fewer individual sphingolipid species showed nominally significant associations with insulin secretion (Supplementary Table S2). Inverse associations with AIRg were observed for the very-long-chain sphingomyelins C28:0 (r = −0.25, P = 0.0003), C30:1(r = −0.27, P < 0.0001), and C30:0 (r = −0.20, P = 0.0044) (r = −0.16, P = 0.024) that had showed positive associations with insulin sensitivity (Supplementary Tables S1, S2). Weaker inverse associations with AIRg were observed for some very-long-chain ceramide species (r = −0.14 to −0.16, P = 0.05 - <0.018) and very-long-chain monohexosylceramide C28:1 (r = −0.16, P = 0.024) (Supplementary Table S2). In contrast, positive associations with AIRg were observed for plasma levels of the long-chain sphingomyelins C18:1 (r = 0.23, P = 0.0009) and C18:0 (r = 0.22, P = 0.0012) that were inversely associated with insulin sensitivity (Supplementary Tables S1, S2).

Unlike the findings regarding insulin sensitivity, most of the associations between individual sphingolipid species and AIRg lost statistical significance after Bonferroni correction for multiple testing, except for the association with very-long-chain sphingomyelins C28:0 and C30:1 (Table 2; Supplementary Table S2). Plasma levels of total very-long-chain sphingomyelins were significantly associated with AIRg (r = −0.27, P < 0.0002). In contrast, total plasma levels of sphingolipids and major species (ceramides, monohexosylceramides, sphingosine) were not significantly associated with insulin secretion (Supplementary Table S2).

**FIGURE 1:**
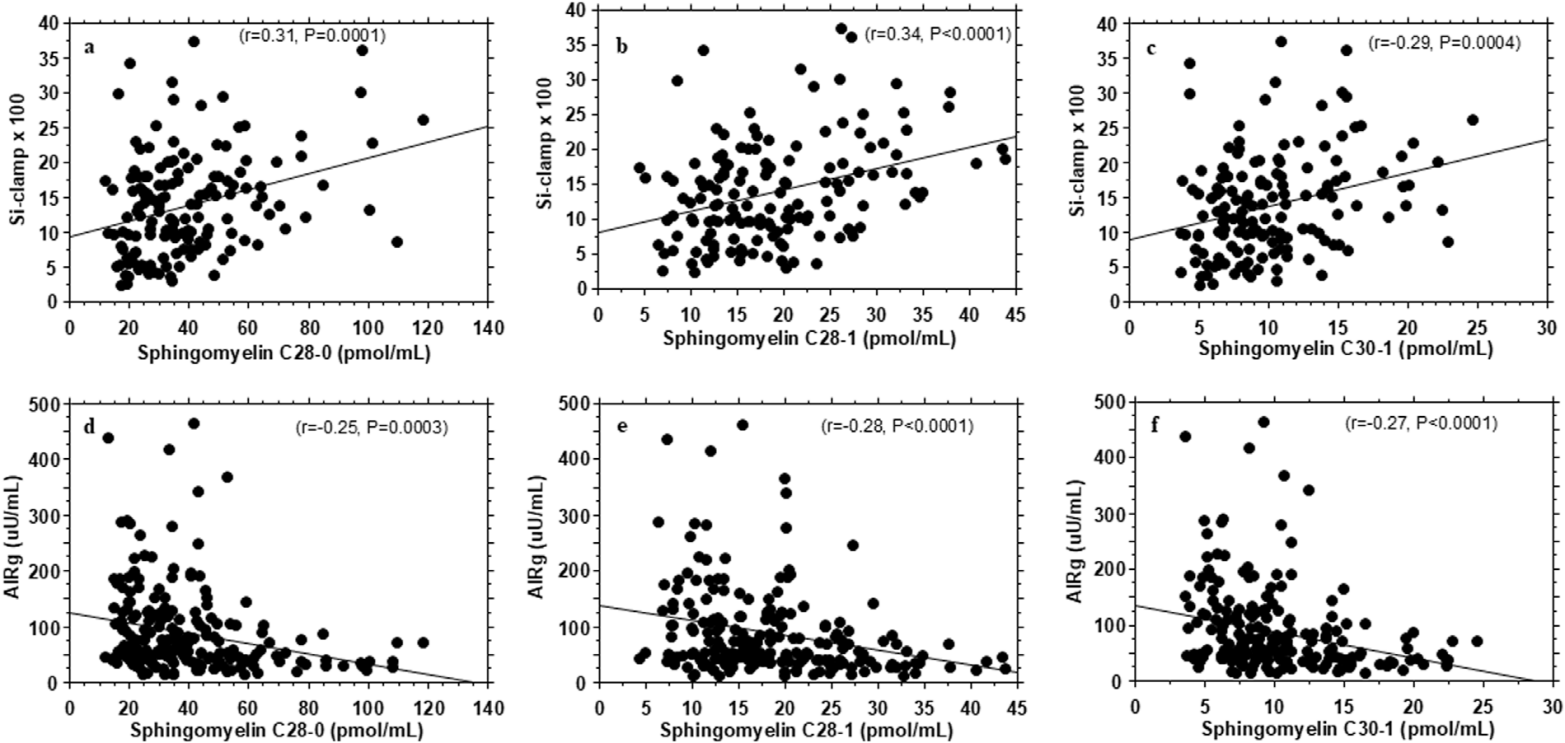
Association of plasma very-long-chain sphingomyelins with insulin sensitivity (Si-clamp {µmol/kg fat-free mass/min.pM^−1^}) **(A–C)** and acute insulin secretory response to i.v. glucose (AIRg) **(D–F)** in healthy, normoglycemic adults.

Plasma levels of very-long-chain sphingomyelins C28:0, C28:1, C30:1 showed concurrent associations with insulin sensitivity (r = 0.31–0.34; P = 0.0004 - <0.0001) and AIRg (r = −0.20 to −0.27; P = 0.004 - <0.0001) (Figure 1; Supplementary Tables S1, S2).

Plasma levels of sphingomyelin C16:0 showed nominal associations with HOMA-IR (r = 0.18, P = 0.038) and HOMA-B (r = 0.23, P = 0.009), as did sphingomyelin C34:0 with HOMA-B (r = 0.18, P = 0.036. However, the associations were not significant after correction for multiple testing. The other sphingolipid species assessed in the present study showed no association with HOMA-IR or HOMA-B.

There were no significant differences by sex or race/ethnicity in the measured sphingolipid species. Further, the observed associations between sphingolipid species and insulin sensitivity and insulin secretion were consistent across race/ethnicity and sex.

### Association with metabolic variables

**FIGURE 2:**
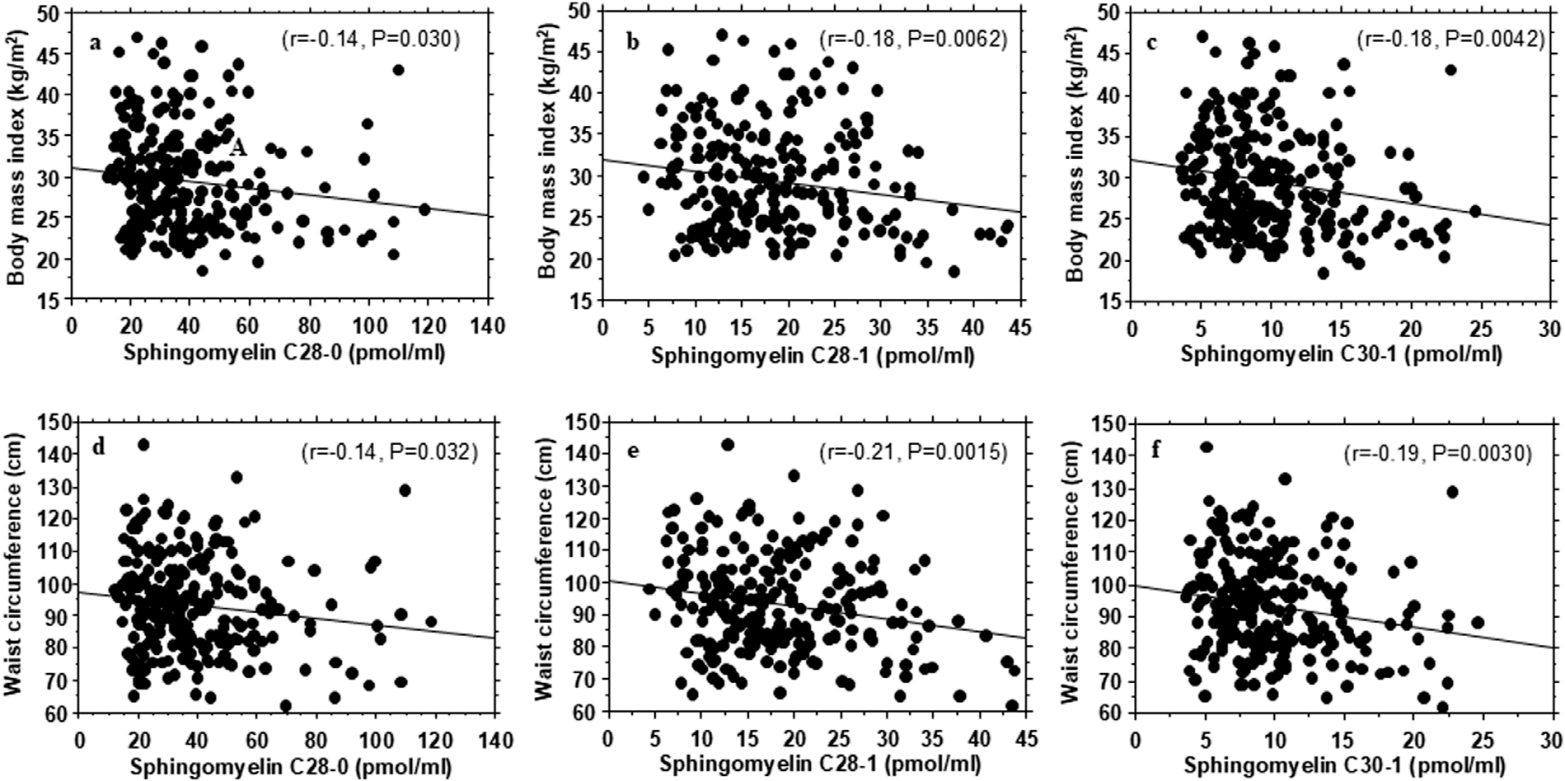
Association of plasma very-long-chain sphingomyelins with body mass index **(A–C)** and waist circumference **(D–F)** in healthy, normoglycemic adults.

Table 3 shows some metabolic correlates of the individual plasma sphingolipid species that exhibited significant associations with insulin sensitivity or insulin secretion. In general, the very-long-chain sphingomyelins and very-long-chain monohexosylceramide C34:0 that were positively associated with insulin sensitivity showed inverse correlations with waist circumference and BMI (Figure 2; Table 3). The very-long-chain monohexosylceramide C34:0 and other very-long-chain sphingomyelin species also were inversely correlated with fasting plasma glucose, 2-h plasma glucose or hemoglobin A1c levels (Table 3).

**TABLE 3:** Correlation of adiposity and glycemic measures with sphingolipid species significantly associated with insulin sensitivity or insulin secretion.

Sphingolipid species	BMI		Waist circumference		FPG		2hrPG		HbA1c	
	r	P	r	P	r	P	r	P	r	P
MHC C34:0	−0.17	0.0075	−0.14	0.026	−0.14	0.028	−0.14	0.026	−0.19	0.0043
SM C28:1	−0.18	0.0062	−0.21	0.0015	0.06	0.38	−0.08	0.21	−0.03	0.66
SM C28:0	−0.14	0.030	−0.14	0.032	0.02	0.74	−0.16	0.017	−0.02	0.77
SM C30:1	−0.18	0.0042	−0.19	0.0030	0.05	0.45	−0.11	0.090	−0.04	0.57
SM C30:0	−0.17	0.010	−0.21	0.0012	−0.09	0.19	−0.23	0.0003	−0.11	0.11
SM C32:0	−0.16	0.011	−0.23	0.0004	−0.13	0.041	−0.13	0.048	−0.17	0.0084
SM C34:0	−0.13	0.046	−0.14	0.038	−0.03	0.66	−0.17	0.010	−0.05	0.44
VLC SM	−0.17	0.0080	−0.19	0.0039	0.01	0.83	−0.16	0.015	−0.04	0.50

FPG, fasting plasma glucose; 2hrPG, two-hour plasma glucose; MHC, monohexosylceramides; SM, sphingomyelin; VLC, very long chain.

## Discussion

Among our healthy, normoglycemic study participants, circulating levels of very-long-chain sphingomyelins correlated positively with insulin sensitivity and inversely with insulin secretion. Remarkably, few of the 58 individual sphingolipid species analyzed in the present study exhibited concurrent associations with insulin sensitivity and insulin secretion, after correction for multiple testing. Directionally, our findings suggest that the association of very-long-chain sphingomyelins with higher insulin sensitivity might be a primary phenomenon that resulted in a compensatory decrease in insulin secretion. It is physiologically less likely that a primary decrease in insulin secretion would trigger an increase in insulin sensitivity. Generally, improvements in insulin action result in decreased insulin demand (secretion).

The positive associations between very-long-chain sphingomyelins with insulin sensitivity suggest that higher levels of those moieties might portend a beneficial glucoregulatory profile, whereas lower levels might connote a less favorable profile. In fact, we found that plasma levels of very-long-chain sphingomyelins were inversely correlated with measures of obesity and glycemia. Thus, participants with higher plasma levels of very-long-chain sphingomyelins were more likely to have higher insulin sensitivity, lower BMI, waist circumference and lower plasma glucose levels, compared with participants with lower levels. Consonant with our findings, lower plasma sphingomyelin levels have been linked to higher risk of developing type 2 diabetes and prediabetes during longitudinal follow-up [15, 32]. Interestingly, weight loss was accompanied by post-operative increases in circulating sphingomyelin levels following gastric bypass surgery [32]. In a previous report, we noted modest differences in plasma levels of some sphingolipid species in people with parental history of type 2 diabetes versus those without a family history of diabetes [32]. In general, circulating levels of ceramide, monohexosylceramide, and sphingosine species were lower and those of sphingomyelin species were higher in individuals with parental history of type 2 diabetes versus people without a family history of diabetes [33].

Very-long-chain fatty acids (>26 carbon chain) are synthesized by ELOVL4 (ELOngation of Very Long chain fatty acid 4) in the endoplasmic reticulum. The very-long-chain fatty acids in sphingolipid classes are acylated by ceramide synthase 3 to a sphingoid backbone to generate very-long-chain ceramides, which are converted to very-long-chain sphingomyelins [1–4, 34–36]. Thus, increased generation of very-long-chain sphingomyelins is associated with decreased abundance of very-long-chain ceramides [34]. Ceramides induce insulin resistance, and higher plasma levels of sphingosine have been shown to predict prediabetes risk [7–10, 15]. Taken together, our finding of a positive correlation between plasma very-long-chain sphingomyelins and whole-body insulin sensitivity is physiologically congruent, as higher sphingomyelins levels would predict a lower abundance of toxic ceramides and sphingosine [34]. Moreover, very-long-chain sphingolipids can regulate insulin action via their specialized role in transmembrane signal transduction [1–10, 37–40]. Notably, exposure to hyperglycemia decreases the levels of very long-chain sphingolipids released by cultured cells [38].

In the present study, the plasma levels of long-chain ceramide C18:1 were inversely associated with insulin sensitivity (i.e., higher levels indicated greater insulin resistance), whereas levels of very-long-chain ceramide C26:0 were positively associated with insulin sensitivity. Remarkably, no other ceramide species with fatty acid carbon chain length C14:0 – C34:0 analyzed in the present study showed significant associations with insulin sensitivity in our normoglycemic cohort. Previous studies that enrolled healthy persons and individuals with obesity and type 2 diabetes had reported similar findings of an inverse association between plasma levels of long-chain dihydroceramides (including C18:0, C20:0, and C22:0) and insulin sensitivity [9, 18].

Elevated ceramide levels were once thought to be uniformly detrimental to metabolic function through induction of insulin resistance, lipotoxicity, and beta-cell dysfunction [5, 6, 8, 10]. However, that view has been modified by reports of heterogeneity in the association between individual ceramide species and insulin sensitivity, based on acyl chain length. Higher levels of long-chain (C16–C22) ceramides are associated with insulin resistance, whereas very-long-chain (C > 22) ceramides are associated with improved insulin sensitivity [40–43]. Thus, our present findings align with the expanded understanding of the complex relationship between ceramides and insulin signaling. A similar dissociation between acyl chain length of ceramides and biological effect has been reported in the cancer literature [42].

The strengths of the present study include the focus on healthy, normoglycemic subjects, which enhances the physiological relevance of our findings. Further, we employed rigorous methodologies for the assessment of insulin sensitivity and acute insulin secretion. We also minimized the risk of false discovery by adjusting the significance of our data for multiple comparisons. Furthermore, it was reassuring that the plasma levels of the selected sphingolipid species measured in the present study were in the same range as values reported in pooled human plasma from healthy men and women in a representative of the US population [44]. One limitation of our study is that we did not include dihydro species of ceramide, monohexosylceramide, sphingomyelins, and other sphingoid base-containing species in our sphingolipid assays. Thus, we were unable to explore associations of insulin sensitivity and secretion with those unmeasured sphingolipid moieties. For example, dihydroceramide desaturase 1-catalyzed conversion of dihydroceramides to ceramides has been suggested to play a role in the pathogenesis of insulin resistance [45]. Thus, inclusion of data on dihydroceramides could have provided a fuller picture on the relationship between sphingolipids and insulin action.

In conclusion, among normoglycemic Black and White American adults, circulating levels of the long-chain sphingolipids (C18:1 ceramide and C18:1 sphingomyelin) were associated inversely with insulin sensitivity, whereas the levels of several very-long-chain sphingolipids were associated positively with insulin sensitivity. We further demonstrate selective association of very-long-chain sphingomyelins with insulin sensitivity, insulin secretion, and adiposity in our normoglycemic adults. Our findings suggest that very-long-chain sphingomyelins may be agents or markers of underlying mechanisms related to optimal metabolic health.

## Data Availability

The original contributions presented in the study are included in the article/Supplementary Material, further inquiries can be directed to the corresponding author.
