# Supplementary material for: Selective association of plasma sphingolipid species with insulin sensitivity and secretion in normoglycemic Black and White American adults
Source: Exp Biol Med (Maywood). 2025 Jun 24;250:10538. doi: 10.3389/ebm.2025.10538 (PMC12234368; doi:10.3389/ebm.2025.10538)
Supplement: Supplementary file 1 [file DataSheet1.PDF]

Supplemental Table S1. Association of Plasma Sphingolipid Species with Insulin Sensitivity

Sphingolipids (pmol/mL)	Mean ± SD	r	P value
**Ceramides (Cer)**			
**C14:0**	12.6 ± 5.40	-0.03	0.76
**C16:0**	65.0 ± 26.9	-0.05	0.58
**C18:1**	6.00 ± 3.48	-0.22	0.0062
**C18:0**	45.4 ± 28.2	-0.10	0.24
**C20:0**	77.9 ± 40.2	-0.03	0.73
**C22:0**	603 ± 311	0.02	0.85
**C24:1**	524 ± 265	0.12	0.15
**C24:0**	1998 ± 994	0.15	0.069
**C26:1**	9.29 ± 5.99	0.16	0.054
**C26:0**	17.4 ± 9.29	0.20	0.014
**C28:1**	0.72 ± 0.73	0.06	0.45
**C28:0**	0.87 ± 1.47	-0.05	0.52
**C30:1**	0.44 ± 0.44	-0.002	0.98
**C30:0**	0.08 ± 0.17	0.05	0.53
**C32:1**	1.43 ± 2.29	-0.07	0.43
**C32:0**	0.35 ± 0.55	-0.01	0.86
**C34:1**	0.67 ± 1.02	-0.07	0.43
**C34:0**	0.14 ± 0.25	-0.02	0.81
**Total Cer**	3363 ± 1478	0.12	0.14
**Total VLC Cer**	4.71 ± 5.88	-0.04	0.64
**Monohexosyl ceramides (MHC)**			
**C14:0**	8.10 ± 4.86	0.22	0.0077
**C16:0**	952 ± 617	-0.02	0.85
**C18:1**	59.2 ± 49.6	-0.19	0.018
**C18:0**	48.0 ± 30.8	-0.07	0.42
**C20:0**	90.2 ± 64.7	0.05	0.57
**C22:0**	1204 ± 767	0.05	0.52
**C24:1**	796 ± 577	-0.001	0.99
**C24:0**	1427 ± 943	0.07	0.42
**C26:1**	13.4 ± 9.89	-0.03	0.68
**C26:0**	7.77 ± 5.57	0.14	0.083
**C28:1**	2.27 ± 1.87	0.20	0.016
**C28:0**	1.43 ± 1.40	0.22	0.0075
**C30:1**	1.14 ± 0.98	0.11	0.21
**C30:0**	7.92 ± 5.93	0.09	0.28
**C32:1**	1.97 ± 2.61	0.07	0.40
**C32:0**	0.35 ± 0.59	0.04	0.61
**C34:1**	0.59 ± 0.50	0.23	0.0048
**C34:0**	0.28 ± 0.28	0.30	0.0002
**Total MHC**	4622 ± 2725	0.03	0.69

**Total VLC MHC**	15.9 ± 10.5	0.17	0.039
**Sphingomyelins (SM)**			
**C14:0**	6271 ± 2145	0.21	0.012
**C16:0**	24287 ± 6963	-0.12	0.15
**C18:1**	6109 ± 2394	-0.23	0.0046
**C18:0**	7374 ± 2526	-0.21	0.011
**C20:0**	6904 ± 2275	-0.01	0.88
**C22:0**	11971 ± 3893	-0.07	0.38
**C24:1**	15654 ± 5090	-0.04	0.60
**C24:0**	8620 ± 3189	-0.06	0.48
**C26:1**	130 ± 51.5	0.02	0.82
**C26:0**	43.9 ± 19.7	-0.02	0.81
**C28:1**	18.5 ± 8.07	0.34	<0.0001
**C28:0**	39.3 ± 20.7	0.31	0.0001
**C30:1**	10.1 ± 4.39	0.29	0.0004
**C30:0**	8.35 ± 3.89	0.32	<0.0001
**C32:1**	1.36 ± 0.74	0.21	0.012
**C32:0**	2.74 ± 1.49	0.35	<0.0001
**C34:1**	0.99 ± 0.51	0.26	0.0016
**C34:0**	1.22 ± 0.61	0.31	0.0001
**Total SM**	87446 ± 23734	-0.09	0.26
**Total VLC SM**	82.5 ± 36.2	0.35	<0.0001
**Sphingosine**	121 ± 54.8	0.11	0.17
**Dh-Sphingosine**	9.89 ± 19.9	0.11	0.19
**S1P**	412 ± 187	0.01	0.91
**Dh-S1P**	70.7 ± 33.2	0.12	0.15
**SPL (Total)**	96045 ± 25667	-0.08	0.36

Dh, dihydro; SIP, sphingosine-1-phosphate; SPL, sphingolipids; VLC, very-long-chain

Supplemental Table S2. Association of Plasma Sphingolipid Species with Insulin Secretion

Sphingolipids (pmol/mL)	Mean ± SD	r	P value
**Ceramides (Cer)**			
**C14:0**	12.6 ± 5.40	-0.07	0.32
**C16:0**	65.0 ± 26.9	-0.01	0.88
**C18:1**	6.00 ± 3.48	0.06	0.40
**C18:0**	45.4 ± 28.2	0.07	0.34
**C20:0**	77.9 ± 40.2	0.01	0.93
**C22:0**	603 ± 311	-0.03	0.72
**C24:1**	524 ± 265	-0.05	0.49
**C24:0**	1998 ± 994	-0.16	0.024
**C26:1**	9.29 ± 5.99	-0.14	0.051
**C26:0**	17.4 ± 9.29	-0.16	0.018
**C28:1**	0.72 ± 0.73	-0.11	0.10
**C28:0**	0.87 ± 1.47	-0.02	0.83
**C30:1**	0.44 ± 0.44	-0.09	0.20
**C30:0**	0.08 ± 0.17	0.02	0.76
**C32:1**	1.43 ± 2.29	-0.02	0.79
**C32:0**	0.35 ± 0.55	-0.07	0.31
**C34:1**	0.67 ± 1.02	-0.002	0.97
**C34:0**	0.14 ± 0.25	-0.02	0.77
**Total Cer**	3363 ± 1478	-0.12	0.084
**Total VLC Cer**	4.71 ± 5.88	-0.04	0.56
**Monohexosyl ceramides (MHC)**			
**C14:0**	8.10 ± 4.86	-0.11	0.11
**C16:0**	952 ± 617	0.01	0.89
**C18:1**	59.2 ± 49.6	0.06	0.37
**C18:0**	48.0 ± 30.8	0.03	0.64
**C20:0**	90.2 ± 64.7	-0.05	0.50
**C22:0**	1204 ± 767	0.001	0.98
**C24:1**	796 ± 577	0.02	0.81
**C24:0**	1427 ± 943	-0.05	0.47
**C26:1**	13.4 ± 9.89	0.05	0.46
**C26:0**	7.77 ± 5.57	0.05	0.46
**C28:1**	2.27 ± 1.87	-0.16	0.024
**C28:0**	1.43 ± 1.40	-0.06	0.42
**C30:1**	1.14 ± 0.98	-0.10	0.15
**C30:0**	7.92 ± 5.93	-0.08	0.23
**C32:1**	1.97 ± 2.61	-0.07	0.31
**C32:0**	0.35 ± 0.59	0.002	0.97
**C34:1**	0.59 ± 0.50	-0.08	0.28
**C34:0**	0.28 ± 0.28	-0.12	0.086
**Total MHC**	4622 ± 2725	-0.01	0.86

**Total VLC MHC**	15.9 ± 10.5	-0.12	0.096
**Sphingomyelins (SM)**			
**C14:0**	6271 ± 2145	-0.12	0.086
**C16:0**	24287 ± 6963	0.12	0.076
**C18:1**	6109 ± 2394	0.23	0.0009
**C18:0**	7374 ± 2526	0.22	0.0012
**C20:0**	6904 ± 2275	0.08	0.26
**C22:0**	11971 ± 3893	0.16	0.018
**C24:1**	15654 ± 5090	0.11	0.13
**C24:0**	8620 ± 3189	0.12	0.089
**C26:1**	130 ± 51.5	0.05	0.50
**C26:0**	43.9 ± 19.7	0.02	0.82
**C28:1**	18.5 ± 8.07	-0.03	0.64
**C28:0**	39.3 ± 20.7	-0.25	0.0003
**C30:1**	10.1 ± 4.39	-0.27	<0.0001
**C30:0**	8.35 ± 3.89	-0.20	0.0044
**C32:1**	1.36 ± 0.74	-0.13	0.057
**C32:0**	2.74 ± 1.49	-0.16	0.024
**C34:1**	0.99 ± 0.51	-0.12	0.086
**C34:0**	1.22 ± 0.61	-0.06	0.39
**Total SM**	87446 ± 23734	0.14	0.038
**Total VLC SM**	82.5 ± 36.2	-0.27	<0.0001
**Sphingosine**	121 ± 54.8	0.09	0.18
**Dh-Sphingosine**	9.89 ± 19.9	0.03	0.72
**S1P**	412 ± 187	0.04	0.55
**Dh-S1P**	70.7 ± 33.2	0.09	0.20
**Total SPL**	96045 ± 25667	0.13	0.070

Dh, dihydro; S1P, sphingosine-1-phosphate; SPL, sphingolipids; VLC, very-long-chain
